# Capacity to Invest Effort as a Predictor of Preference for Digital Mental Health Interventions Over Psychotherapy: Cross-Sectional Study Using an Ecological Digital Screening Tool

**DOI:** 10.2196/77802

**Published:** 2025-10-20

**Authors:** Tomer Savir, Amit Baumel

**Affiliations:** 1 Department of Community Mental Health, Faculty of Social Welfare and Health Sciences, University of Haifa, Abba Khoushy Ave 199, Haifa, 3498838, Israel, 972 8288496 ext 4, 972 8288723

**Keywords:** preferences, capacity to invest, effort, distress, severity, mental health, digital interventions, screening, internet, digital health

## Abstract

**Background:**

Research typically shows a higher preference for professionally led face-to-face mental health interventions over digital ones. It remains unclear in which circumstances digital self-help tools are preferred. To address this gap, it is important to examine user characteristics that may help predict when digital interventions are more desirable, ultimately guiding their design to enhance engagement and appeal.

**Objective:**

This study aims to examine how distress severity and capacity to invest effort relate to intervention preferences, using an ecological assessment of individuals seeking to receive feedback on their mental health.

**Methods:**

A comprehensive digital mental health screening tool with automated feedback was developed and advertised on social media. The sample comprised 684 adult participants aged 18 to 82 years who opted to complete the screening to receive feedback on their mental health state. Participants completed questionnaires measuring general psychological distress, depression, generalized anxiety, and demographics. The Kessler Psychological Distress Scale–6 was used as the primary measure for distress. Participants were also presented with questions measuring capacity to invest effort and preferences for a professional therapist versus digital self-help tools and for psychotherapy versus a mobile app. The effectiveness of distress, capacity to invest effort, and background characteristics in predicting preferences (a professional vs digital self-help tools; psychotherapy vs a mobile app) was examined using hierarchical linear regressions. The distributions of dichotomized preferences were plotted against distress and capacity to invest effort for transparent visualization.

**Results:**

A hierarchical linear regression found that distress, capacity to invest, and currently being in psychotherapy significantly predicted preference for a professional versus digital self-help tools. Distress (β=.25, 95% CI .18-.32; *P*<.001) and capacity to invest effort (β=.23, 95% CI .16-.30; *P*<.001) were the strongest predictors, with similar effect size. The model explained 20% of the variance in preference, with the capacity to invest effort uniquely contributing 5%. Most participants experiencing distress with low capacity (158/239, 66.1%) preferred digital self-help tools, whereas most participants experiencing distress with high capacity (147/243, 60.5%) favored a professional. Similar results were obtained when using the Patient Health Questionnaire–4 as an alternative distress measure. Capacity to invest effort remained significant (β=.18, 95% CI .10-.26; *P*<.001) when predicting a preference for psychotherapy versus a mobile app, while distress was not significant (β=−.03, 95% CI −.10 to .05; *P*=.51).

**Conclusions:**

This study highlights that the preference for digital interventions is driven by a reduced capacity to invest effort in an intervention. Attempts to reduce the mental health treatment gap through digital interventions should focus on optimizing the effort elicited by users to improve desirability and engagement.

## Introduction

### Background

Digital mental health interventions have been recognized as a promising avenue for reducing the mental health treatment gap by offering a more accessible alternative to traditional care [1,2], which is often limited by barriers such as stigma, costs, availability of clinicians, and time or distance constraints [3]. A variety of digital mental health tools have been developed over the past decade for diverse mental health purposes [4,5] and are seeing increased global availability [6].

The desirability of digital interventions has been studied across different target populations, such as community samples [7-9], students [10-12], and older adults [13]. Preferences are usually examined among adult nonpatients, assessed for depression, anxiety, or nonspecific psychological distress [11,12,14-16], often using online settings [7,8,10-12,14,15,17]. Despite the increased prevalence and accessibility of digital interventions, and the accumulated evidence indicating their efficacy [18], research consistently shows a higher preference for traditional professionally led face-to-face interventions compared to digital self-help tools [7,8,10,12,16,17,19]. For example, March et al [8] reported that 85.7% of participants who were recruited online from a community sample preferred face-to-face services over digital interventions. Similarly, Klein and Cook [7] found a comparable preference, with 77.1% of participants favoring in-person services. Despite the converging findings, it still remains unclear in which circumstances digital self-help tools are preferred over receiving psychotherapy from a professional.

Various factors were examined as predictors of preference for digital interventions. For example, technology confidence and low extraversion personality [8], belief in the effectiveness of digital treatments [10], stigmatized beliefs toward mental illness [7], and the appeal of their availability [15] and low costs [14] were found to predict a higher preference for digital interventions. While informative, these findings are limited in guiding stakeholders with the purpose of increasing digital interventions’ desirability, uptake, and continued engagement. It is therefore also important to examine user preferences capable of informing developers on how to design digital interventions that are more likely to be desired by individuals seeking mental health care. The current severity of individuals’ psychological distress and their capacity to invest effort in a mental health intervention reflect both their need for mental health care and their actual capability to pursue it. While distress severity has sometimes been explored as a predictor of intervention preferences, the capacity to invest effort has not yet been studied as a predictor of preferences. Examining the effect of these dynamic factors on intervention preferences may further our understanding of intervention desirability and help inform the appropriate design of digital interventions tailored to such needs and circumstances.

### Psychological Distress

If left untreated, mental illness can worsen in severity over time [20,21]. Even though an early decision to seek help may be pivotal for treatment prognosis [22,23], individuals commonly withhold seeking help until their symptoms become severe and disrupt their lives [24-27].

Research regarding the effect of illness severity on preferences toward digital interventions presents inconsistent findings and differing metrics. Using depression as an example, a study using an online research panel found that depression severity was negatively associated with acceptability of a digital intervention (β=−.29), indicating that higher levels of depression were associated with a lower preference for digital interventions [15]. In contrast, another study using an online survey found depression severity to be the second strongest positive predictor of intentions to use digital interventions as a substitute for face-to-face settings (β=.23), meaning that higher levels of depression were associated with a higher preference for digital interventions [28]. In other studies, no association was found between symptom or distress severity and intervention preferences among primary care clinic visitors who were later approached online [9] or college students recruited online [11]. Importantly, a naturalistic study in which patients with depression could choose their treatment modality found that those with greater symptom severity preferred in-person therapy over supported digital interventions [29].

Online interventions are often perceived by both health professionals and laypeople to be inadequate for treating severe symptoms and are instead deemed acceptable mostly for mild or moderate conditions [30], despite research supporting their efficacy in cases of severe illness [31]. Thus, it may be assumed that individuals experiencing high levels of distress may be more likely to pursue interventions perceived as better suited to contend with intense difficulties, namely, those led by professionals. Considering that individuals often delay seeking help until their symptoms are severe and distressing, this may help explain the consistent finding that most help seekers prefer face-to-face interventions.

### Capacity to Invest Effort

Individuals contemplating seeking help are inevitably mindful of their current capacity to invest effort in a mental health intervention. Seeking psychotherapy from a mental health professional is usually considered to be relatively demanding in terms of costs, time, and effort commitment and typically requires the client to be able to travel to a therapist’s location on a fixed weekly schedule—requirements that some help seekers may find difficult or unfeasible to meet [3].

The capacity to invest effort in a mental health intervention is likely linked to the formation of intervention preferences upon seeking mental health care. Different intervention modalities vary in the volume of effort and investment required, appealing to individuals with corresponding capabilities. Notably, help seekers concerned about cost or time requirements tend to prefer digital interventions [9,16,17], as they are often perceived, advantageously, as requiring less effort and investment [17,32]. Indeed, the extent to which an intervention can be seamlessly integrated into daily routines in terms of time investment and motivation can affect engagement with it [33]. As a result, the development of digital mental health tools takes into consideration the degree of effort they elicit from users to increase engagement [34,35].

Therefore, it may be assumed that due to their merits of being recognized as smaller scale and more easily accessible and used, digital self-help tools may appeal to individuals whose current circumstances reflect a limited capacity to invest. Understanding whether the current capacity to invest effort is related to preferences toward digital self-help tools is paramount, as it may help guide the development and design of digital interventions in ways that would appeal to individuals with low capacity, thus increasing their desirability and the continued engagement with them.

### Objectives

This study aimed to examine how individuals’ current level of distress and capacity to invest effort in a mental health intervention relate to their preference for a professional versus digital self-help tools. These factors were examined using a comprehensive mental health screening tool developed for this study, enabling an ecological assessment of individuals’ current circumstances. The most relevant point in time for examining individuals’ intervention preferences and how they relate to their current circumstances is when they reach out to understand their mental health state and ponder whether to seek help, before the stage of referral to appropriate services. It was hypothesized that high levels of distress and capacity to invest effort would each predict a tendency to prefer help from a professional as opposed to a preference for digital self-help tools.

## Methods

### Design, Participants, and Recruitment Procedure

We conducted an online cross-sectional study. As we aimed to detect the unique contributions of distress and capacity to invest effort in a model containing numerous predictors, and considering that no previous literature exists regarding effort capacity and possible interactions, we conducted an a priori sample size analysis using G*Power (version 3.1; Heinrich-Heine-University Düsseldorf) [36]. Assuming small effect sizes (*f*²=0.02), α=.05, and power=0.80, the required sample size for our planned model was approximately N=400. A sensitivity check indicated that approximately N=300 would still be able to detect a slightly larger effect size (*f*²=0.025). Accordingly, we targeted recruitment of 300‐400 participants, aiming for 400 as a conservative goal.

Participants were recruited between March 13, 2024, and August 6, 2024, through a Facebook (Meta Platforms Inc) advertising campaign, relevant social media mental health groups, and digital banners shared through WhatsApp (Meta Platforms Inc). The advertisement invited individuals to take a self-examination to receive comprehensive feedback on their mental health status and information about courses of action. Individuals who chose to take the self-examination accessed a digital screening tool. They were presented with a short description of the tool, provided informed consent, answered questions aimed to verify their understanding of the screening tool’s purpose, and then completed questionnaires measuring different mental health difficulties. After the screening was completed, participants received automated, detailed personalized feedback and were asked to review the screening process.

Hebrew-speaking adults aged 18 years or older with computer or smartphone access were eligible to use the screening tool. The study sample consisted only of participants who completed all the study questionnaires, up to and including the demographic questionnaire, as this ensured we would be able to test the research hypotheses and demographically characterize the participants. Participants who did not complete the screening process or completed it in an unreasonably short time were excluded from the sample.

This study is reported in accordance with the STROBE (Strengthening the Reporting of Observational Studies in Epidemiology) guidelines (Checklist 1) for cross-sectional studies [37] and the CHERRIES (Checklist for Reporting Results of Internet E-Surveys) guidelines (Checklist 2) [38].

### Mental Health Screening and Feedback Tool

A comprehensive digital screening battery was developed using the survey platform Qualtrics (Silver Lake), providing users with automated personalized feedback regarding their mental health status and suggestions for courses of action. The battery included questionnaires screening for numerous mental health difficulties and a set of questions examining users’ preferences toward different interventions and their capacity to invest effort in a mental health intervention. During the screening, all participants completed questionnaires measuring general psychological distress, depression, generalized anxiety, and demographics. All participants were also asked questions measuring their intervention preferences and capacity to invest. Additional questionnaires measuring various mental health conditions relevant to participants’ reported difficulties were presented, based on responses to preliminary questions inspired by the DIAMOND (Diagnostic Interview for Anxiety, Mood, and OCD and Related Neuropsychiatric Disorders) structured diagnostic interview [39]. The selected mental health questionnaires met the highest clinical assessment standards for both development and validation [40]. After completing the screening, participants received automated, personalized feedback corresponding with their assessment results. They were then offered the option to download an automatically generated file containing their screening results. The questionnaires and feedback presented are available to view on the Open Science Framework [41].

### Measurements

The Kessler Psychological Distress Scale–6 (K6) [42] is a self-report questionnaire measuring nonspecific psychological distress using 6 items on a 5-point Likert scale ranging from 0 to 4. It was developed to identify severe distress using a cutoff of 13 [43]. A moderate distress category was later defined with a cutoff of 5 [44]. The K6 was found useful in detecting nonspecific mental illness and general severity and is often used in large-scale population health assessments [44,45]. It demonstrates strong psychometric properties, including high internal consistency (Cronbach α=0.89), high discriminant validity, and strong predictive values [42,43]. In this study, it was found to have similarly high internal consistency (Cronbach α=0.86).

The Patient Health Questionnaire–4 (PHQ-4) [46] is a self-report questionnaire measuring depression and anxiety symptoms using 4 items on a 4-point Likert scale ranging from 0 to 3. It is considered a viable measure of psychological distress [46] and was used in this study as an alternative distress measure to the K6 for a sensitivity analysis, using the relevant items from the Patient Health Questionnaire–9 [47] and the Generalized Anxiety Disorder Scale–7 [48] which were administered to all participants during the screening. PHQ-4 scores can be categorized to differentiate between levels of distress using established clinical cutoffs. A score of 0 to 2 reflects no distress, a score of 3 to 5 reflects mild distress, a score of 6 to 8 reflects moderate distress, and a score of 9 to 12 reflects severe distress [46]. The PHQ-4 demonstrated high internal consistency (Cronbach α=0.85), along with high sensitivity and specificity in the detection of various mental health disorders [46]. In this study, it was found to have high internal consistency (Cronbach α=0.78).

The participants’ current capacity to invest effort in a mental health intervention was measured by 2 items developed for this study. Participants were asked to rate their readiness to invest effort in mental health interventions in terms of time and travel for the intervention to be successful. The items were rated on a scale ranging from 1=“I would not be able to invest even a minute” and “I would not be able to travel at all to receive the intervention” to 100=“I would be able to invest an hour every day” and “I would be able to travel a 30 min’ drive distance even two times every week.*”* The 2 items were averaged to form a capacity to invest effort score ranging from 1 to 100, with higher scores indicating greater current capacity to invest effort. The scale was found to have a modest reliability coefficient of 0.64, using the Spearman-Brown coefficient for 2-item scales [49]. It is important to note that 2-item scales tend to produce lower reliability coefficients [50-52], often lower than the conventional threshold of 0.7 [53-55]—even for well-established measures such as Generalized Anxiety Disorder Scale–2 [56]. Nevertheless, given the modest reliability score, we also conducted a sensitivity analysis, presenting the results for each item separately (Multimedia Appendix 1).

The participants were asked how they would like to address the matter of their current mental health by rating their degree of preference for a professional or digital self-help tools using a scale ranging from 1=“I’m interested in receiving digital tools that would allow me to improve it myself” to 100=“I’m interested in receiving help from a professional.” Higher ratings on this scale indicated a stronger preference for a professional, whereas lower ratings indicated a stronger preference for digital self-help tools.

The preference between psychotherapy and a mobile app was used as an alternative measure to assess whether individuals’ choices between professional support and digital self-help tools align with their preferences for the corresponding conventional delivery formats available today. Participants were presented with short descriptions of 2 mental health interventions accompanied by illustrative pictures: weekly psychotherapy and mobile apps. Following the descriptions, they were asked to rate the degree to which they would want to receive each intervention on a scale ranging from 1 to 7. A preference difference score between the 2 ratings was calculated, ranging from −6 to 6, where scores higher than 0 indicated a higher preference for psychotherapy and scores lower than 0 indicated a higher preference for mobile apps. For the purpose of dichotomous preference classification, participants who rated the 2 interventions equally were consequently asked to indicate which intervention they preferred or whether they had no preference between them.

In a demographic questionnaire, the participants reported their gender, age, education, relationship status, religion, and religiosity. They additionally specified whether they have been in psychotherapy in the past and whether they are currently in psychotherapy.

### Ethical Considerations

This study is part of a preregistered PhD proposal, which included a full description of the study and its hypotheses, and was approved on August 29, 2023, by the University of Haifa Graduate Studies Authority. The study protocol was approved by the institutional research ethics committee of the University of Haifa (approval 422/23). Participants provided informed consent and were able to opt out or discontinue their participation at any stage of the screening. Study data were anonymous. Participants were offered an automatic download of their screening feedback upon completion.

### Statistical Analyses

The effectiveness of K6 and the capacity to invest effort in predicting preference toward a professional versus digital self-help tools were examined using a hierarchical linear regression. The degree of preference toward a professional versus digital self-help tools was defined as the outcome variable. Independent variables were entered into the regression in 4 consecutive steps, entering the model based on statistical significance. The first step included background characteristics, adding variables such as gender, age, education, relationship status, religion, religiosity, having been in psychotherapy in the past, and currently being in psychotherapy, using the stepwise method (forward selection). In the second and third steps, K6 and capacity to invest effort were each added as single focal variables (K6 in the second step and capacity in the third step). Finally, the fourth step added interaction variables, created between predictors that were found to be significant in previous steps, using the stepwise method (forward selection). K6 and capacity to invest effort were mean-centered before creating the interaction variables to reduce multicollinearity in the regression model [57]. In the regression analysis, the demographic variables gender, education, relationship status, religion, and religiosity were condensed and dichotomized according to the distribution and scarcity of certain categories in our sample. The same statistical approach was then used to examine how K6 and capacity to invest effort predicted preference for psychotherapy versus a mobile app, using the preference difference score as the outcome variable. To examine the consistency of the results, we conducted sensitivity analyses in relevant cases using the same statistical approach (Multimedia Appendix 2). All statistical analyses were conducted in SPSS (version 29; IBM Corp).

### Graphical Illustrations

For transparent visualization of the predictive value of K6 and the capacity to invest effort on preference toward a professional versus digital self-help tools, the predicted variable was dichotomized by defining a score of 40 or lower as a preference for digital self-help tools and a score of 60 or higher as a preference for a professional. To examine the consistency of this dichotomization method and for comprehensiveness, we also examined classifications of 70-30 and 80-20 and presented them in additional analysis. The distribution of preferences was plotted against K6 and the capacity to invest effort. K6 was categorized based on established clinical cutoffs [44], whereas the capacity to invest effort was categorized into high or low capacity based on the median sample capacity. The same approach was also used to graphically illustrate how K6 and capacity to invest effort predict preference for psychotherapy versus a mobile app, with preference difference scores above 0 indicating a preference for psychotherapy and scores below 0 indicating a preference for a mobile app.

## Results

### Overview

**Figure 1.**
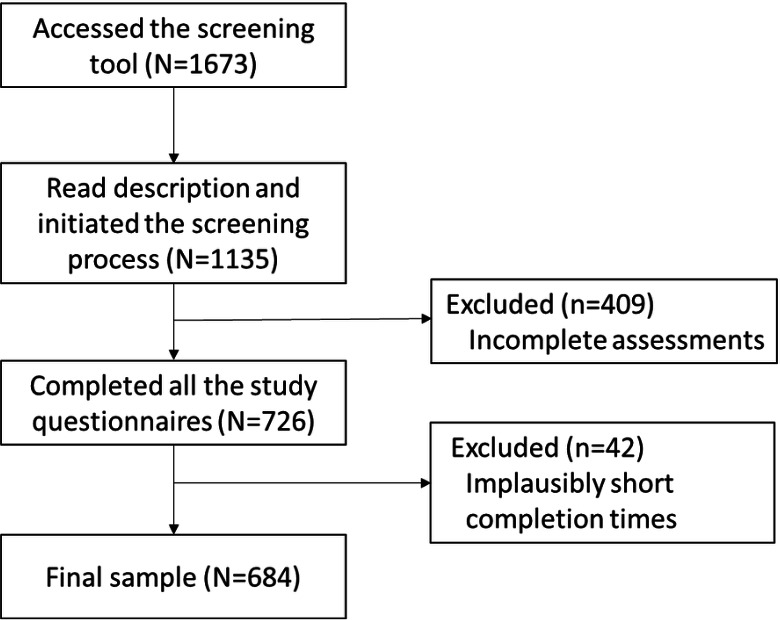
Participant flowchart showing how the final study sample was derived from all individuals who accessed the screening tool, through completion of the screening and completion time exclusion criterion.

The participant flowchart is presented in Figure 1. The final sample consisted of 684 adults (aged 18‐82 y; mean 41.04, SD 13.49 y).

Background characteristics and descriptive statistics of current circumstances and preferences among the study sample are presented in Table 1. Most participants were women (502/684, 73.4%), Jewish (652/684, 95.3%), had an academic education (439/684, 64.2%), and had past experience with psychotherapy (464/684, 67.8%). The sample was predominantly distressed (K6: mean 11.67, SD 5.12; PHQ-4: mean 5.81, SD 3.07) and with relatively high capacity to invest effort (mean 64.67, SD 25.04). Preferences between a professional and digital self-help tools were approximately evenly split (mean 47.20, SD 33.30).

**Table 1.** Background characteristics, current circumstances, and preference-related descriptive statistics of the study sample (N=684).

Variable	Value
Dichotomous variables, n (%)	
Gender^a^	
Woman	502 (73.4)
Man	172 (25.1)
Education	
Academic	439 (64.2)
Nonacademic	245 (35.8)
Relationship status	
In a relationship	388 (56.7)
Not in a relationship	296 (43.3)
Religion	
Jewish	652 (95.3)
Other religion	32 (4.7)
Religiosity	
Secular	469 (68.6)
Religious	215 (31.4)
Been in psychotherapy in the past	
Yes	464 (67.8)
No	220 (32.2)
Currently in psychotherapy	
Yes	227 (33.2)
No	457 (66.8)
Continuous variables, mean (SD)	
Age (y)	41.04 (13.49)
K6^b^	11.67 (5.12)
PHQ-4^c^	5.81 (3.07)
Capacity to invest effort	64.67 (25.04)
Degree of preference for a professional versus digital self-help tools	47.20 (33.30)
Preference difference between psychotherapy and a mobile app^d^	0.41 (3.11)

a On the basis of 674 participants who chose to identify as a binary gender choice.

b K6: Kessler Psychological Distress Scale–6.

c PHQ-4: Patient Health Questionnaire–4.

d Difference between preference ratings of psychotherapy and a mobile app; positive values represent a stronger preference for psychotherapy.

### Preference for a Professional Versus Digital Self-Help Tools

The hierarchical linear regression result for predicting the degree of preference for a professional versus digital self-help tools using background characteristics, K6, and capacity to invest effort is presented in Table 2. The regression model included only the significant variables produced by the stepwise method. The first model containing background characteristics contributed 10% to the explained variance of preference. The addition of K6 to the regression model uniquely contributed an additional 6% to the explained variance, and the addition of capacity to invest effort uniquely contributed an additional 5%. The final regression model contained 3 significant predictors: K6, the capacity to invest effort, and being currently in psychotherapy. K6 (β=.25, 95% CI .18-.32; *P*<.001) and capacity to invest effort (β=.23, 95% CI .16-.30; *P*<.001) were found to be the stronger predictors with a similar effect size.

**Table 2.** Hierarchical linear regression predicting degree of preference for a professional versus digital self-help tools^a^.

Variable	Model 1^b^: background characteristics			Model 2^c^: K6^d^			Model 3^e^: capacity to invest effort		
	β (95% CI)		*P* value	*β* (95% CI)		*P* value	β (95% CI)		*P* value
Age (y)	−*.10* (−0.17 to –0.03)^f^		.008	−.05 (−.12 to .03)		.220	−.05 (−.12 to .02)		.19
Education	*.09 *(.02 to .16)		.01	.04 (−.03 to .11)		.242	.04 (−.03 to .11)		.26
Been in psychotherapy in the past	.*12* (.04 to .20)		.003	*.08 *(.01 to .16)		.037	.06 (−.01 to .14)		.11
Currently in psychotherapy	.*21* (.13 to .29)		<.001	*.18 *(.10 to .25)		<.001	*.13* (.05 to .20)		.001
K6^g^	—^h^		—	*.26* (.19 to .34)		<.001	*.25* (.18 to .32)		<.001
Capacity to invest effort^g^	—		—	—		—	*.23* (.16 to .30)		<.001

a Positive β values indicate that higher predictor values are associated with a stronger preference for a professional versus digital self-help tools.

b In model 1, the adjusted *R*², *R*² change, and *R*² change *P* value are 0.10, 0.10, and <.001, respectively.

c In model 2, the adjusted *R*², *R*² change, and *R*² change *P* value are 0.16, 0.06, and <.001, respectively.

d K6: Kessler Psychological Distress Scale–6.

e In model 3, the adjusted *R*², *R*² change, and *R*² change *P* value are .20, .05, and <.001, respectively.

f Italicized β values represent significant predictors.

g Variable was mean-centered to reduce multicollinearity in the regression model.

h Em dashes indicate predictors added in subsequent models.

**Figure 2.**
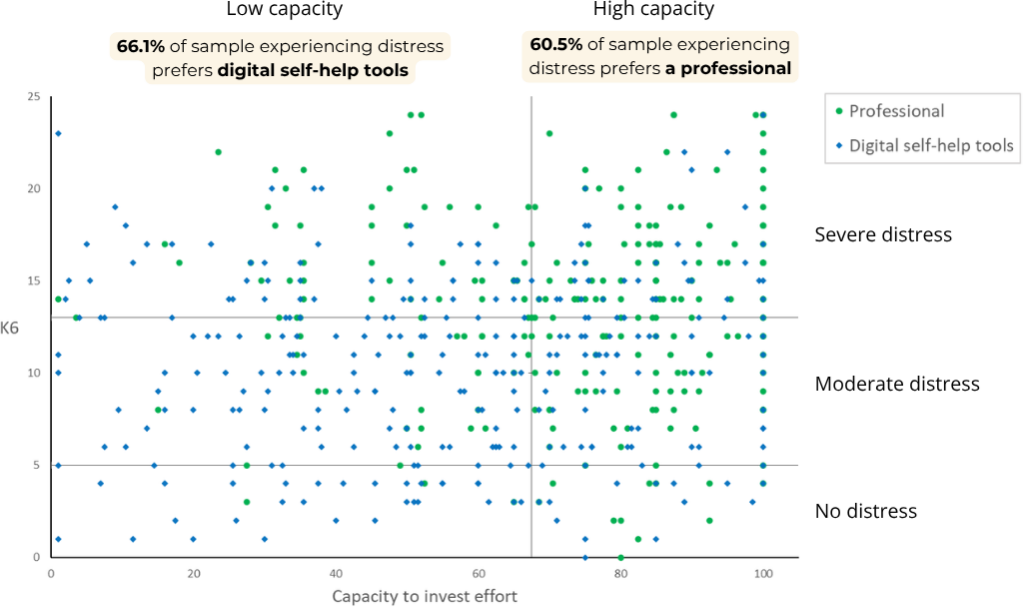
Scatterplot of preferences for a professional versus digital self-help tools by Kessler Psychological Distress Scale (K6) score and capacity to invest effort, indicating a preference reversal among participants experiencing distress with low or high capacity to invest effort (n=538).

Figure 2 displays the scatterplot of preferences for a professional versus digital self-help tools by K6 scores and the capacity to invest effort. Table 3 summarizes the distribution of preferences across 6 distinct categories defined by K6 and the capacity to invest effort levels. As illustrated, participants experiencing distress (moderate or severe distress) with a low level of capacity to invest effort showed a higher frequency of preference for digital self-help tools (158/239, 66.1%) over a professional (81/239, 33.9%). This tendency was reversed for participants experiencing distress with a high level of capacity to invest effort who mostly preferred a professional (147/243, 60.5%) over digital self-help tools (96/243, 39.5%).

**Table 3.** Preferences for a professional versus digital self-help tools by categories defined by Kessler Psychological Distress Scale (K6) and capacity to invest effort levels (n=538).

Category		Preferences, n (%)^a^	
		A professional	Digital self-help tools
Low capacity			
Not distressed		3 (9.4)	29 (90.6)
Distressed (total)		81 (33.9)	158 (66.1)
Moderate distress		27 (22)	96 (78)
Severe distress		54 (46.6)	62 (53.4)
High capacity			
Not distressed		11 (45.8)	13 (54.2)
Distressed (total)		147 (60.5)	96 (39.5)
Moderate distress		53 (50)	53 (50)
Severe distress		94 (68.6)	43 (31.4)

a Percentages are based on the distribution of preferences within each category.

### Preference for Psychotherapy Versus a Mobile App

The hierarchical linear regression result for predicting preference difference between psychotherapy and a mobile app using background characteristics, K6, and capacity to invest effort is presented in Table 4. The first model containing background characteristics contributed 10% to the explained variance of preference. The addition of capacity to invest effort to the regression model uniquely contributed an additional 3% to the explained variance. The final regression model contained 4 significant predictors: capacity to invest effort, being currently in psychotherapy, been in psychotherapy in the past, and age. While the capacity to invest effort (β=.18, 95% CI .10-.26; *P*<.001) was found to be a significant predictor, K6 (β=−.03, 95% CI −.10 to .05; *P*=.51) was not significant and was not included in the regression model.

**Table 4.** Hierarchical linear regression predicting preference difference between psychotherapy and a mobile app^a^.

Variable	Model 1^b^: background characteristics			Model 2^c^: capacity to invest effort		
	β (95% CI)		*P* value	β (95% CI)		*P* value
Age (y)	*−.10* (−.18 to −.02)^d^		.007	*−.10 *(−.17 to −.03)		.006
Been in psychotherapy in the past	*.17 *(.09 to .25)		<.001	*.16 *(.08 to .23)		<.001
Currently in psychotherapy	*.17* (.09 to .25)		<.001	*.14* (.06 to .21)		.001
Capacity to invest effort^e^	—^f^		—	*.18* (.10 to .26)		<.001

a Positive β values indicate that higher predictor values are associated with a stronger preference for psychotherapy versus a mobile app.

b In model 1, the adjusted *R*², *R*² change, and *R*² change *P* value are .10, .10, and <.001, respectively.

c In model 2, the adjusted *R*², *R*² change, and *R*² change *P* value are .13, .03, and <.001, respectively.

d Italicized β values represent significant predictors.

e Variable was mean-centered to reduce multicollinearity in the regression model.

f Em dashes indicate predictors added in subsequent models.

**Figure 3.**
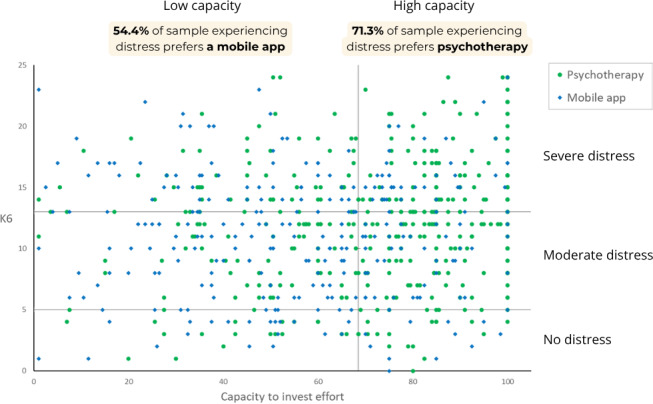
Scatterplot of preferences for psychotherapy versus a mobile app by Kessler Psychological Distress Scale–6 (K6) score and capacity to invest effort, indicating a similar preference reversal among participants experiencing distress with low or high capacity to invest effort (N=645).

Figure 3 displays the scatterplot of preferences for a mobile app versus psychotherapy by K6 scores and capacity to invest effort. Table 5 summarizes the distribution of preferences across the 6 categories defined by K6 and the capacity to invest effort levels. Figure 3 and Table 5 demonstrate a similar preference distribution pattern to the one observed in Figure 2 and Table 3. Among participants experiencing distress (moderate or severe), those with low capacity to invest effort more frequently preferred a mobile app (156/287, 54.4%) over psychotherapy (131/287, 45.6%); however, participants experiencing distress with high capacity to invest effort more frequently preferred psychotherapy (209/293, 71.3%) over a mobile app (84/293, 28.7%).

**Table 5.** Preferences for psychotherapy versus a mobile app by categories defined by Kessler Psychological Distress Scale–6 (K6) and capacity to invest effort levels (N=645).

Category		Preferences, n (%)^a^	
		Psychotherapy	Mobile app
Low capacity			
Not distressed		20 (51.3)	19 (48.7)
Distressed (total)		131 (45.6)	156 (54.4)
Moderate distress		64 (42.7)	86 (57.3)
Severe distress		67 (48.9)	70 (51.1)
High capacity			
Not distressed		15 (57.7)	11 (42.3)
Distressed (total)		209 (71.3)	84 (28.7)
Moderate distress		93 (68.9)	42 (31.1)
Severe distress		116 (73.4)	42 (26.6)

a Percentages are based on the distribution of preferences within each category.

### Sensitivity Analyses

The 2 items comprising the capacity to invest effort scale were also examined separately. Each item individually showed similar patterns to those presented in Table 2 (Multimedia Appendix 1). PHQ-4 was examined as an alternative distress variable. The results and distribution patterns using PHQ-4 were similar to those obtained by using K6 (Multimedia Appendix 2). We examined further classification methods to dichotomize preference for a professional versus digital self-help tools, differing from the 60‐40 classification method used. The observed effects and distribution patterns were maintained for classifications of 70‐30 (n=458) and 80‐20 (n=352). The results for these classifications are provided in Multimedia Appendix 3.

## Discussion

### Principal Findings

This study examined the effects of distress severity and capacity to invest effort on mental health intervention preferences, using an ecological assessment of individuals’ current circumstances and preferences. Both distress and capacity to invest effort emerged as the strongest predictors of preferences for a professional versus digital self-help tools, showing positive correlations and similar effect sizes. In terms of capacity to invest effort, the pattern found was that most participants experiencing distress with a *low level of capacity effort* favored digital self-help tools, whereas most participants experiencing distress with a *high level of capacity effort* favored a professional. A similar preference distribution was found for participants experiencing distress based on their level of capacity to invest effort when examining the preference between a mobile app and psychotherapy as an alternative preference measure. While distress was not significant in the prediction of this preference choice, the capacity to invest effort remained a significant predictor, highlighting the robustness of its effect on intervention preferences.

Our findings indicate the importance of individuals’ current capacity to invest effort on their preferences toward different modes of mental health care, as lower levels of capacity were associated with a preference toward digital self-help tools. This suggests that individuals are sensitive to the perceived effort an intervention requires [32] and are then drawn to interventions that correspond with their current capacity to invest effort. This insight is crucial for designing digital interventions aiming to offer an alternative to traditional modes of care, underscoring the importance of optimizing the effort that interventions demand from users [35] to appeal to those with low levels of capacity. Furthermore, implementation and dissemination of new and existing digital interventions may benefit from emphasizing the minimal effort required to use them to increase their desirability among target populations.

In regard to the role of distress in relation to intervention preferences, a positive association was found between distress severity and a preference for a professional versus digital self-help tools. The association was consistent across different severity measures but inconsistent across preference choices, as no significant association was found between distress and the preference difference between psychotherapy and a mobile app. This further accentuates the inconsistency observed in previous studies of distress and symptom severity in predicting intervention preferences [9,11,15,28,29]. It may be that distress motivates individuals to prefer professional assistance; however, both psychotherapy and a mobile app were presented in depth as established mental health interventions—potentially blurring differences in perceived suitability.

In addition to distress and capacity to invest effort, currently being in psychotherapy was also found to be a significant predictor, associated with a stronger preference toward a professional. The finding that currently participating in a professionally led mental health intervention was associated with a preference for this type of intervention is consistent with previous studies showing a link between prior treatment experience and preference for in-person care [10]. Interestingly, currently being in psychotherapy was found to be a weaker predictor of preferences compared with distress and capacity to invest. It is unclear, however, whether this implies that being in psychotherapy reflects a genuine preference for that mode of intervention or alternatively reflects a lack of awareness of digital alternatives [58,59] and their advantages [15].

The setting used in this study indicates the potential of using an ecological mental health screening to examine intervention preferences at the point when individuals are first inquiring about their difficulties. The online domain is typically the first method by which individuals seek mental health information and care [60], and online screening has several advantages, such as easy access to a large number of people, time and cost efficiency, and an increased capability to disclose personal difficulties [61,62]. This approach can also serve as a key gateway for offering different types of interventions based on individuals’ preferences and current capacity to invest effort.

### Limitations and Considerations for Future Research

This study has several limitations. First, our sample comprised a relatively high proportion of women and participants with academic education. While this is consistent with gender differences observed in established digital screening tools [63] and with reports showing a high proportion of the Israeli population with tertiary education [64], patterns may differ in other locations. Second, individuals who choose to complete the screening might have a higher innate willingness to use digital tools. However, participants’ past or current experience with digital interventions was not documented in the screening and thus not examined for possible effects on preferences, which could be examined in future research.

Third, the capacity to invest effort was measured in this study using a 2-item scale. Our sensitivity analysis yielded no significant differences between the responses to the 2 items and our main analysis; however, the results are thus influenced by both time investment and unwillingness to travel. Therefore, future studies should continue to examine individuals’ capacity to invest effort in different ways and how this may influence outcomes. The scale used in this study demonstrated modest reliability but yielded robust results. This demonstrates that short scales are sometimes adequate for detecting empirical correlations despite lower reliability [52] and that, when in the context of a comprehensive screening scale, brevity may justify its lower psychometrics [51]. Nonetheless, future studies aiming to assess the capacity to invest effort may develop alternative scales or consider expanding the scale used in this study, adding more nuanced items to be used in contexts that allow broader assessment without concerns about participant fatigue.

### Conclusions

This study highlighted the role of individuals’ current circumstances in relation to their intervention preferences. It was shown that a preference for digital self-help tools is driven by a reduced capacity and willingness to invest effort in an intervention. Individuals low on capacity might greatly benefit from digital interventions designed to induce minimal effort and burden on their daily life [34] and are likely to prefer such interventions over traditional face-to-face ones. Attempts to reduce the mental health treatment gap through digital interventions should focus on optimizing the effort required [35] to enhance engagement and desirability, providing effective alternatives for individuals with limited capacity who still need support.

## Supplementary material

10.2196/77802 Multimedia Appendix 1 Results for individual items measuring capacity to invest effort.

10.2196/77802 Multimedia Appendix 2 Results using the Patient Health Questionnaire–4 to measure distress.

10.2196/77802 Multimedia Appendix 3 Different classifications of preference for a professional versus digital self-help tools.

10.2196/77802 Checklist 1 STROBE statement—items recommended for inclusion in reports of cross-sectional studies.

10.2196/77802 Checklist 2 CHERRIES guidelines.

## Abbreviations

CHERRIES: Checklist for Reporting Results of Internet E-Surveys
DIAMOND: Diagnostic Interview for Anxiety, Mood, and OCD and Related Neuropsychiatric Disorders
K6: Kessler Psychological Distress Scale–6
PHQ-4: Patient Health Questionnaire–4
STROBE: Strengthening the Reporting of Observational Studies in Epidemiology
