# Supplementary material for: Capacity to Invest Effort as a Predictor of Preference for Digital Mental Health Interventions Over Psychotherapy: Cross-Sectional Study Using an Ecological Digital Screening Tool
Source: J Med Internet Res. 2025 Oct 20;27:e77802. doi: 10.2196/77802 (PMC12536998; doi:10.2196/77802)
Supplement: Multimedia Appendix 1 [file jmir-v27-e77802-s001.pdf]

Multimedia Appendix – Results For Individual Items Measuring Capacity To Invest Effort

The hierarchical linear regression results for predicting degree of preference for a professional vs self-help tools using background characteristics, K6, and capacity to invest effort (time) is presented in Table S3. As the Table presents, capacity to invest effort (time) maintained a similar direction and significance to the two-item capacity to invest effort results presented in Table 2.

Table S3. Hierarchical linear regression predicting degree of preference for a professional vs self-help tools[a].

	Model 1: background characteristics			Model 2: K6			Model 3: capacity to invest effort (time)		
Variable	β	95% CI	*P* value	β	95% CI	*P* value	β	95% CI	*P* value
Age	*-.10*[b]	-.17 to -.03	.008	-.05	-.12 to .03	.220	-.06	-.13 to .02	.126
Education	*.09*	.02 to .16	.014	.04	-.03 to .11	.242	.04	-.03 to .11	.324
Been in psychotherapy in the past	*.12*	.04 to .20	.003	*.08*	.01 to .16	.037	.07	.00 to .15	.069
Currently in psychotherapy	*.21*	.13 to .29	<.001	*.18*	.10 to .25	<.001	*.15*	.08 to .23	<.001
K6[c]	—[d]	—	—	*.26*	.19 to .34	<.001	*.24*	.17 to .32	<.001
Capacity to invest effort (time)[c]	—	—	—	—	—	—	*.16*	.09 to .23	<.001
Adjusted R^2	.10			.16			.18		
R^2 change	*.10*			*.06*			*.03*		
R^2 change *P* value	<.001			<.001			<.001		

[a] Positive β values indicate that higher predictor values are associated with a stronger preference for a professional vs digital self-help tools.
[b] Italicized β values represent significant predictors.
[c] Variable was mean-centered to reduce multicollinearity in the regression model.
[d] Em dashes indicate predictors added in subsequent models.

The hierarchical linear regression result for predicting degree of preference for a professional vs self-help tools using background characteristics, K6, and capacity to invest

effort (travel) is presented in Table S4. Capacity to invest effort (travel) had a similar effect size to the two-item capacity to invest effort, maintaining a similar direction and significance to the results presented in Table 2.

Table S4. Hierarchical linear regression predicting degree of preference for a professional vs self-help tools[a].

	Model 1: background characteristics			Model 2: K6			Model 3: capacity to invest effort (travel)		
Variable	β	95% CI	*P* value	β	95% CI	*P* value	β	95% CI	*P* value
Age	*-.10*[b]	-.17 to -.03	.008	-.05	-.12 to .03	.220	-.04	-.11 to .03	.310
Education	*.09*	.02 to .16	.014	.04	-.03 to .11	.242	.05	-.02 to .12	.188
Been in psychotherapy in the past	*.12*	.04 to .20	.003	*.08*	.01 to .16	.037	.06	-.01 to .14	.102
Currently in psychotherapy	*.21*	.13 to .29	<.001	*.18*	.10 to .25	<.001	*.13*	.05 to .20	.001
K6[c]	—[d]	—	—	*.26*	.19 to .34	<.001	*.27*	.20 to .34	<.001
Capacity to invest effort (travel) [c]	—	—	—	—	—	—	*.22*	.15 to .29	<.001
Adjusted R^2	.10			.16			.20		
R^2 change	*.10*			*.06*			*.04*		
R^2 change *P* value	<.001			<.001			<.001		

[a] Positive β values indicate that higher predictor values are associated with a stronger preference for a professional vs digital self-help tools.

[b] Italicized β values represent significant predictors.

[c] Variable was mean-centered to reduce multicollinearity in the regression model.

[d] Em dashes indicate predictors added in subsequent models.

This is a Multimedia Appendix to a full manuscript entitled Capacity to Invest Effort as a Predictor of Preference for Digital Mental Health Interventions Over Psychotherapy: Cross-Sectional Study Using an Ecological Digital Screening Tool, published in the J Med Internet Res. For full copyright and citation information see http://dx.doi.org/10.2196/jmir.77802
