# Supplementary material for: Capacity to Invest Effort as a Predictor of Preference for Digital Mental Health Interventions Over Psychotherapy: Cross-Sectional Study Using an Ecological Digital Screening Tool
Source: J Med Internet Res. 2025 Oct 20;27:e77802. doi: 10.2196/77802 (PMC12536998; doi:10.2196/77802)
Supplement: Multimedia Appendix 2 [file jmir-v27-e77802-s002.pdf]

Multimedia Appendix – Results Using The Patient Health Questionnaire-4 To Measure Distress

The hierarchical linear regression results for predicting degree of preference for a professional vs self-help tools using background characteristics, Patient Health Questionnaire-4 (PHQ-4), and capacity to invest effort is presented in Table S1. The final regression model contained five significant predictors: capacity to invest effort, PHQ-4, currently in psychotherapy, been in psychotherapy in the past, and age, with effect sizes that are similar to the results presented in Table 2.

Table S1. Hierarchical linear regression predicting degree of preference for a professional vs self-help tools[a].

	Model 1: background characteristics			Model 2: PHQ-4			Model 3: capacity to invest effort		
Variable	β	95% CI	*P* value	β	95% CI	*P* value	β	95% CI	*P* value
Age	*-.10*[b]	-.17 to -.03	.008	*-.08*	-.15 to -.01	.035	*-.08*	-.15 to -.01	.029
Education	*.09*	.02 to .16	.014	.06	-.01 to .13	.102	.06	-.01 to .13	.114
Been in psychotherapy in the past	*.12*	.04 to .20	.003	*.10*	.02 to .18	.012	*.08*	.00 to .15	.045
Currently in psychotherapy	*.21*	.13 to .29	<.001	*.18*	.10 to .25	<.001	*.13*	.05 to .20	.002
PHQ-4[c]	—[d]	—	—	*.22*	.14 to .29	<.001	*.21*	.14 to .28	<.001
Capacity to invest effort[c]	—	—	—	—	—	—	*.23*	.16 to .30	<.001
Adjusted R^2	.10			.14			.19		
R^2 change	*.10*			*.04*			*.05*		
R^2 change *P* value	<.001			<.001			<.001		

[a] Positive β values indicate that higher predictor values are associated with a stronger preference for a professional vs digital self-help tools.

[b] Italicized β values represent significant predictors.

[c] Variable was mean-centered to reduce multicollinearity in the regression model.

[d] Em dashes indicate predictors added in subsequent models.

Figure S1 displays the scatter plot of preferences for a professional vs digital self-help tools by PHQ-4 scores and capacity to invest effort. Table S2 then summarizes the distribution of preferences across six distinct categories defined by PHQ-4 and capacity to invest effort levels. Figure S1 and Table S2 show the effects of both PHQ-4 and capacity to invest effort in predicting participants' preference, with similar distribution trends to the ones observed in Figure 2 and Table 3.

Figure S1. Scatterplot of preferences for a professional versus digital self-help tools by Patient Health Questionnaire-4 (PHQ-4) score and capacity to invest effort, indicating a preference reversal among participants experiencing distress with low or high capacity to invest effort (n=538).

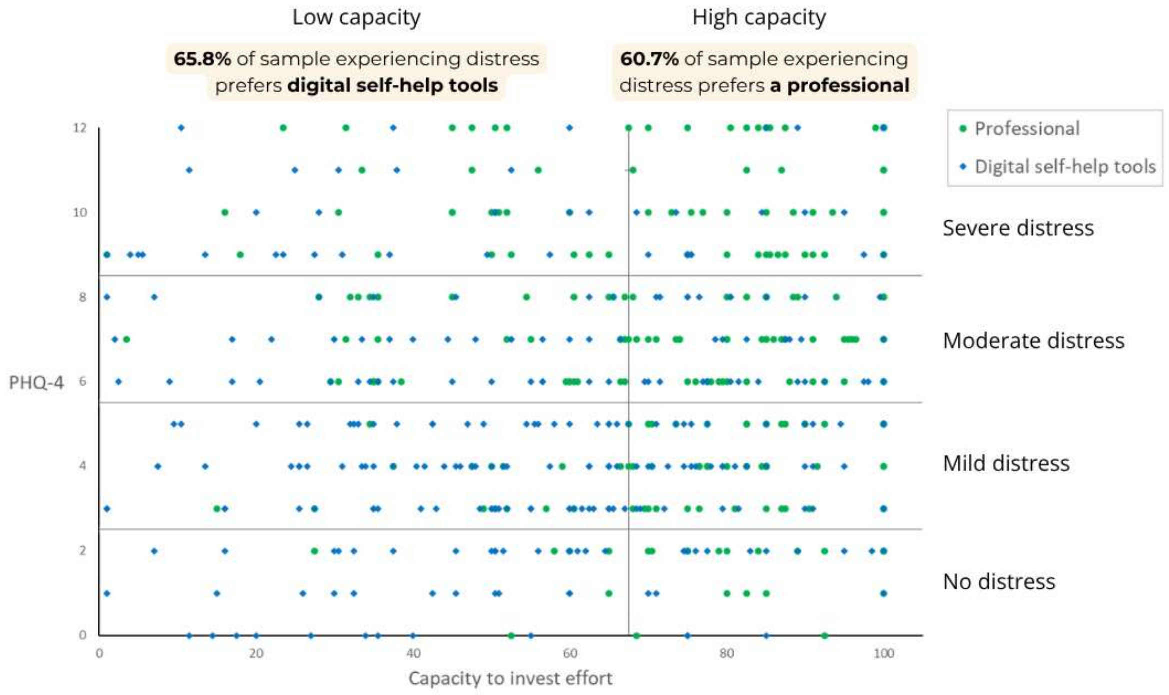

Table S2. Preferences for a professional vs digital self-help tools by categories defined by PHQ-4 and capacity to invest effort levels (n=538).

Category		Preferences, n (%)[a]	
		A professional	Digital self-help tools
Low capacity	Not distressed	6 (14.0)	37 (86.0)
	Distressed (total)	78 (34.2)	150 (65.8)
	mild distress	16 (16.0)	84 (84.0)
	moderate distress	35 (47.3)	39 (52.7)
	severe distress	27 (50.0)	27 (50.0)
High capacity	Not distressed	19 (50.0)	19 (50.0)
	Distressed (total)	139 (60.7)	90 (39.3)
	mild distress	46 (54.1)	39 (45.9)
	moderate distress	49 (60.5)	32 (39.5)
	severe distress	44 (69.8)	19 (30.2)

[a] Percentages are based on the distribution of preferences within each category.

This is a Multimedia Appendix to a full manuscript entitled Capacity to Invest Effort as a Predictor of Preference for Digital Mental Health Interventions Over Psychotherapy: Cross-Sectional Study Using an Ecological Digital Screening Tool, published in the J Med Internet Res. For full copyright and citation information see http://dx.doi.org/10.2196/jmir.77802
