# Supplementary material for: Capacity to Invest Effort as a Predictor of Preference for Digital Mental Health Interventions Over Psychotherapy: Cross-Sectional Study Using an Ecological Digital Screening Tool
Source: J Med Internet Res. 2025 Oct 20;27:e77802. doi: 10.2196/77802 (PMC12536998; doi:10.2196/77802)
Supplement: Multimedia Appendix 3 [file jmir-v27-e77802-s003.pdf]

<u>Multimedia Appendix – Different Classifications of Preference for a Professional versus Digital Self-Help Tools</u>

Figure S2 and Figure S3 display the scatter plot of preferences for a professional vs digital self-help tools by K6 scores and capacity to invest effort – using a 70-30 and 80-20 preference classification method respectively. Table S5 and Table S6 summarize the distribution of both preference classifications across six distinct categories defined by K6 and capacity to invest effort levels. As the figures and tables illustrate, the effects of both K6 and capacity to invest effort in predicting participants' preference and the distribution trends are maintained when progressing from a 60-40 (Figure 2 and Table 3) to 70-30 and 80-20 classification methods.

Figure S2. Scatterplot of preferences for a professional versus digital self-help tools by Kessler Psychological Distress Scale (K6) score and capacity to invest effort using 70-30 classification, indicating a maintained preference reversal among participants experiencing distress with low or high capacity to invest effort (n=458).

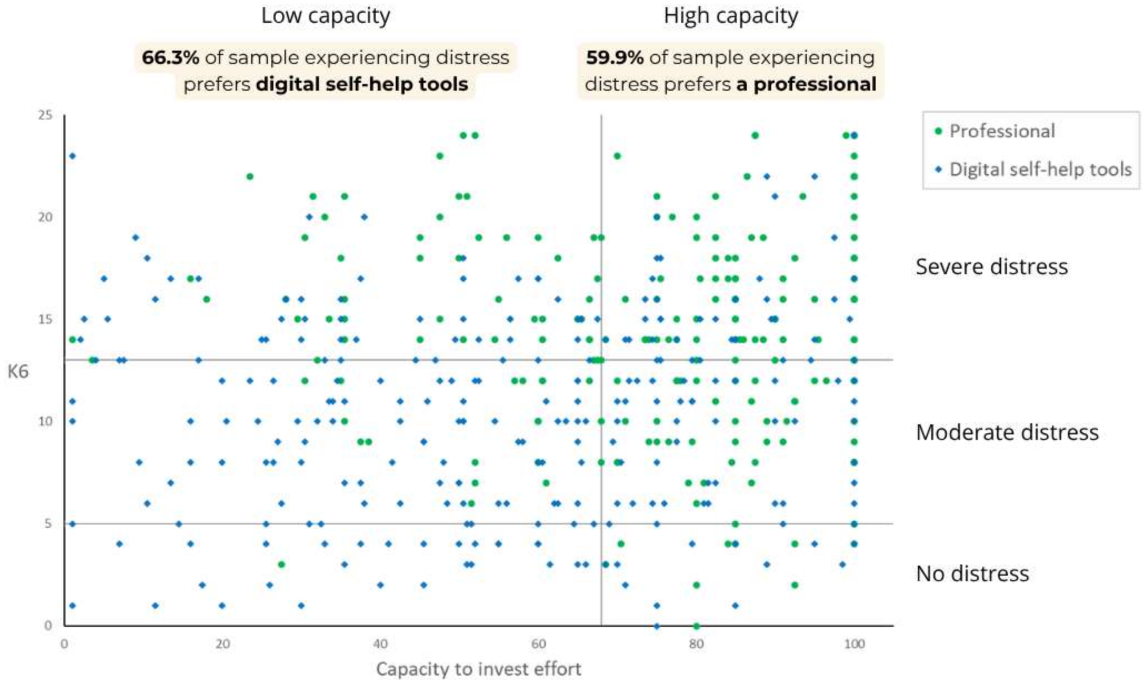

Table S5. Preferences for a professional vs digital self-help tools by categories defined by K6 and capacity to invest effort levels using 70-30 classification (n=458).

Category		Preferences, n (%)[a]	
		A professional	Digital self-help tools
Low capacity	Not distressed	1 (3.6)	27 (96.4)
	Distressed (total)	68 (33.7)	134 (66.3)
	moderate distress	19 (19.0)	81 (81.0)
	severe distress	49 (48.0)	53 (52.0)
High capacity	Not distressed	9 (42.9)	12 (57.1)
	Distressed (total)	124 (59.9)	83 (40.1)
	moderate distress	42 (49.4)	43 (50.6)
	severe distress	82 (67.2)	40 (32.8)

[a] Percentages are based on the distribution of preferences within each category.

Figure S3. Scatterplot of preferences for a professional versus digital self-help tools by Kessler Psychological Distress Scale (K6) score and capacity to invest effort using 80-20 classification, indicating a maintained preference reversal among participants experiencing distress with low or high capacity to invest effort (n=352).

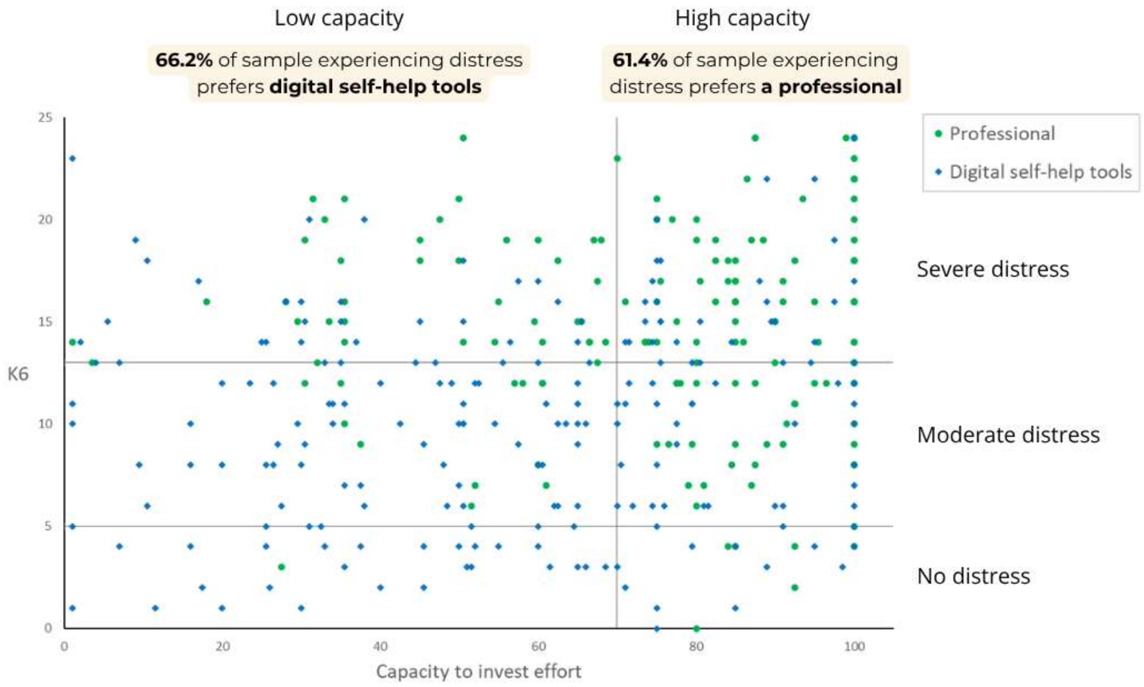

Table S6. Preferences for a professional vs digital self-help tools by categories defined by K6 and capacity to invest effort levels using 80-20 classification (n=352).

Category		Preferences, n (%)[a]	
		A professional	Digital self-help tools
Low capacity	Not distressed	1 (3.7)	26 (96.3)
	Distressed (total)	51 (33.8)	100 (66.2)
	moderate distress	12 (15.8)	64 (84.2)
	severe distress	39 (52.0)	36 (48.0)
High capacity	Not distressed	6 (37.5)	10 (62.5)
	Distressed (total)	97 (61.4)	61 (38.6)
	moderate distress	30 (50.8)	29 (49.2)
	severe distress	67 (67.0)	32 (33.0)

[a] Percentages are based on the distribution of preferences within each category.

This is a Multimedia Appendix to a full manuscript entitled Capacity to Invest Effort as a Predictor of Preference for Digital Mental Health Interventions Over Psychotherapy: Cross-Sectional Study Using an Ecological Digital Screening Tool, published in the J Med Internet Res. For full copyright and citation information see http://dx.doi.org/10.2196/jmir.77802
