# Supplementary material for: Capacity to Invest Effort as a Predictor of Preference for Digital Mental Health Interventions Over Psychotherapy: Cross-Sectional Study Using an Ecological Digital Screening Tool
Source: J Med Internet Res. 2025 Oct 20;27:e77802. doi: 10.2196/77802 (PMC12536998; doi:10.2196/77802)
Supplement: Checklist 2 [file jmir-v27-e77802-s005.pdf]

Multimedia Appendix – Checklist for Reporting Results of Internet E-Surveys (CHERRIES)

Item Category	Checklist Item	Explanation
**Design**		
	Describe survey design	We conducted an online cross-sectional study, targeting individuals who chose to take a self-examination in order to receive comprehensive feedback on their mental health status and information about courses of action.
**IRB (Institutional Review Board) approval and informed consent process**		
	IRB approval	This study is part of a pre-registered PhD proposal that included a full description of the study and its hypotheses, approved on 29.8.2023 by the University of Haifa Graduate Studies Authority. The study protocol was approved by the Institutional Review Board of University of Haifa (Approval No. 422/23).
	Informed consent	Participants were first presented a short description of the screening tool and the study, including information regarding expected completion time and data confidentiality. They then provided informed consent, and answered questions aimed to verify their understanding of the screening tool's purpose, before initiating the screening. Participants were able to opt out or discontinue their participation at any stage of the screening.
	Data protection	Anonymity was maintained by avoiding the collection of personally identifiable information, including IP addresses. Data was stored securely on the Qualtrics platform and was accessible only to the research team.
**Development and pre-testing**		
	Development and testing	The screening tool was developed by the research team, inspired by established digital mental health screening services. A digital mental health screening expert was consulted during development. The tool was reviewed by mental health professionals and tested by laypersons for comprehensiveness, usability and comprehension. Feedback from the reviews informed revisions before the tool was advertised.
**Recruitment process and description of the sample having access to the questionnaire**		
	Open survey versus closed survey	The survey was open, with a public link shared digitally through Facebook and WhatsApp.
	Contact mode	Recruitment was conducted digitally using a public survey link shared through Facebook and WhatsApp.
	Advertising the survey	The study was advertised via a Facebook advertising campaign, posting in relevant social media mental health groups, and digital banners shared through personal and professional WhatsApp networks. The advertisement invited individuals to take a mental health self-examination as part of a study on preferences in order to receive comprehensive feedback on their mental health status and information about courses of action.
**Survey administration**		
	Web/E-mail	The screening tool was administered via the online platform Qualtrics, accessed by using a public link.
	Context	Links to the screening tool were shared across social media mental health groups, Facebook advertising, and relevant

		WhatsApp networks, allowing the study invitation to reach individuals with interest in examining their mental health state.
	Mandatory/voluntary	Participation was completely voluntary. Participants were able to opt out or discontinue their participation at any stage of the screening.
	Incentives	After screening completion, participants received automated detailed personalized feedback regarding their mental health state and courses of action. They were then offered the option to download an automatically generated file containing their screening results.
	Time/Date	Data collection was conducted between March 13, 2024 and August 6, 2024.
	Randomization of items or questionnaires	No randomization of items or questionnaires was used.
	Adaptive questioning	Questionnaires measuring certain mental health conditions were presented or omitted based on participants' responses to preliminary questions gauging relevant difficulties. The feedback following screening completion was personalized based on participants' responses during the screening.
	Number of Items	The number of items varied across different participants due to adaptive questioning. Complete participations contained a minimum of 92 items. Items and questionnaires were spread so that no more than one questionnaire was displayed per screen (page).
	Number of screens (pages)	The number of screens (pages) varied across different participants due to adaptive questioning. Complete participations contained a minimum of 34 screens (pages), each containing a minimal number of items to reduce bloat.
	Completeness check	Response for most items was required to proceed with the screening, ensuring data completeness for providing the personalized mental health feedback and for analyzing the research questions.
	Review step	Participants could review and change their answers using the "Back" button at several steps of the screening but not throughout. Changing answers was not possible once the feedback was generated.
**Response rates**		
	Unique site visitor	Unique visitors were not tracked digitally in order to completely maintain anonymity and the privacy of participants' mental health information. An item at the beginning of the screening asked participants whether they used the screening tool before.
	View rate (Ratio of unique survey visitors/unique site visitors)	Not Applicable.
	Participation rate (Ratio of unique visitors who agreed to participate/unique first survey page visitors)	A total of 1673 individuals accessed the screening tool via the advertised public link and 1135 of them gave informed consent and initiated the screening process, indicating a participation rate of 67.8%.
	Completion rate (Ratio of users who finished the survey/users who agreed to participate)	Of 1135 participants who initiated the screening, 726 completed the process, indicating a completion rate of 64.0%
**Preventing multiple entries from the same individual**		
	Cookies used	Cookies were not used to track participants.

	IP check	IP addresses were not collected nor used to track participants.
	Log file analysis	An item at the beginning of the screening asked participants whether they used the screening tool before. In addition, participants with implausibly short completion times were excluded from the analysis. Few participants indicated having used the tool before, all of whom were counted among those excluded due to implausibly short completion times.
	Registration	Not applicable, as this was an open survey.
**Analysis**		
	Handling of incomplete questionnaires	The final sample consisted only of participants who completed all the study questionnaires, up to and including the demographic questionnaire, as this ensured we would be able to test the research hypotheses and demographically characterize the participants.
	Questionnaires submitted with an atypical timestamp	Participants with implausibly short completion times were excluded from the analysis.
	Statistical correction	No statistical correction methods were used. All statistical analyses were conducted in SPSS v.29.

This is a Multimedia Appendix to a full manuscript entitled Capacity to Invest Effort as a Predictor of Preference for Digital Mental Health Interventions Over Psychotherapy: Cross-Sectional Study Using an Ecological Digital Screening Tool, published in the J Med Internet Res. For full copyright and citation information see http://dx.doi.org/10.2196/jmir.77802
